# Prediction and characterization of a novel hemocyanin-derived antimicrobial peptide from shrimp *Litopenaeus vannamei*

**DOI:** 10.1007/s00726-018-2575-x

**Published:** 2018-05-04

**Authors:** Shen Yang, He Huang, Fan Wang, Jude Juventus Aweya, Zhihong Zheng, Yueling Zhang

**Affiliations:** Department of Biology and Guangdong Provincial Key Laboratory of Marine Biotechnology, School of Science, Shantou University, Shantou, 515063 Guangdong China

**Keywords:** *Litopenaeus vannamei*, Hemocyanin, Prediction, Antimicrobial peptide

## Abstract

Hemocyanin, the multifunctional glycoprotein in the hemolymph of invertebrates, can generate various antimicrobial peptides (AMPs). Given the rising interest in the use of natural therapeutic agents such as AMPs, alternative and more efficient methods for their generation are being explored. In this work, free online software was first applied to predict the generation of antimicrobial peptides from the large subunit of *Litopenaeus vannamei* hemocyanin. Twenty potential antimicrobial peptides ranging from 1.5 to 1.9 kDa were predicted, five of which had α-helical structures and were selected for antibacterial activity testing. The results indicated that these five peptides had antibacterial activity against seven different bacteria. Of the five peptides, one peptide, designated L1, had the strongest antibacterial activity against both Gram-negative and Gram-positive bacteria. Moreover, CD and NMR data showed that L1 had both α-helical and β-turns structural composition, and that these structures were essential for L1’s antibacterial activity. Furthermore, SEM analysis revealed that peptide L1 had broad-spectrum activity against both Gram-positive and Gram-negative bacteria, as it could destroy the bacterial cell walls and kill the bacteria. Thus, L1 is a very potent antimicrobial peptide that can be exploited and used in antibacterial therapeutics.

**Electronic supplementary material:**

The online version of this article (10.1007/s00726-018-2575-x) contains supplementary material, which is available to authorized users.

## Introduction

Unlike vertebrates which have both innate and adaptive immune systems, the penaeid shrimp is an invertebrate arthropod with only an innate immune system for protection against pathogens (Bachere et al. 2004). Antimicrobial peptides (AMPs) are a major part of the innate immune defense mechanism and have been extensively studied in the past 20 years. AMPs have broad-spectrum activity against different organisms including Gram-positive and Gram-negative bacteria (Zhu et al. 2014; Dolashka et al. 2011; Zhuang et al. 2015), as well as fungi, viruses, parasites, and some cancer cells (Choi and Lee 2014; Bulmer et al. 2010; Riciluca et al. 2012; Badani et al. 2014; Liu et al. 2012; Adade et al. 2013; Deng et al. 2016).

Recently, some AMPs derived from high molecular weight proteins have been identified. For instance, Kondori et al. (2011) found that the peptide HLopt2 (20–31) derived from the N-terminal of human lactoferrin had antimicrobial activity against five pathogenic fungi, including *Cryptococcus neoformans*, *Candida albicans*, *Candida krusei*, *Candida kefyr* and *Candida parapsilosis*. Nguyen et al. (2010) reported that AMPs could be derived from the C-terminal α-helical structure of human chemokines macrophage inflammatory protein-3α (MIP-3α/CCL20), interleukin-8 (IL-8), neutrophil activating protein-2 (NAP-2) and thrombocidin-1 (TC-1), all of which have direct antimicrobial activities. Zhang et al. (2013) have also shown that AJHba peptides from *Anguilla japonica* had strong antibacterial activities, which was similar to hemoglobin alpha in the liver. A 19-mer peptide, HP (2-20), derived from the N-terminal region of *Helicobacter pylori* ribosomal protein L1 (RPL1), was shown to possess broad-spectrum antimicrobial activities (Park and Hahm 2005). Similarly, hemocyanin, the most abundant protein in arthropods, is reported to also generate various AMPs (Lee et al. 2003; Destoumieux-Garzon et al. 2001; Wen et al. 2016).

Although there are a number of studies on antimicrobial peptides, there is still limited information on the mechanisms by which these peptides are produced and their mode of action. For these reasons, there is the need to further research and explore the field of antimicrobial peptides and their activity. To be able to leverage on the benefits of AMPs, more efficient methods of generation and/or production are being explored. With the advancement in bioinformatics tools and platforms, several antimicrobial peptides could be accurately and efficiently predicted using laboratory-generated data and the relevant software (Loose et al. 2006; Fjell et al. 2011; Kai et al. 2005). Moreover, since the main features of antimicrobial peptides are known, these bioinformatics tools can be exploited for novel antimicrobial peptides prediction and screening.

Here, we first applied a bioinformatics approach and were able to predict and successfully identified 20 potential AMPs from hemocyanin of *Litopenaeus vannamei*, 5 of which were further explored. Screening of the peptides showed that peptide L1 had the strongest antibacterial activity against both Gram-positive and Gram-negative bacteria. Further analysis of the antimicrobial mechanism of L1 using nuclear magnetic resonance and scanning electron microscopy revealed that L1 had an α-helical and β-turns structure, and that these structures were important for its antibacterial activity. Our results indicate that hemocyanin can generate AMPs with strong antimicrobial activity.

## Experimental

### Prediction of antibacterial peptides from *L. vannamei* hemocyanin

Three online softwares, Antibacterial peptides (AntiBP) Server, Collection of Antimicrobial Peptides (CAMP) Server and Antimicrobial Peptide Calculator and Predictor (APD2), were applied to predict antibacterial peptides from *L. vannamei* hemocyanin large subunit (GenBank: CAB85965.1). Of the softwares used, AntiBP Server (http://www.imtech.res.in/raghava/antibp/index.html) could predict antibacterial peptides based on protein sequence, with the prediction done by using support vector machine (SVM)-based method via peptide sequences. CAMP (http://www.camp. bicnirrh.res.in) contains information on the conserved sequence signatures, which were captured as patterns and hidden Markov models (HMMs) from 1386 AMPs divided into 45 families. On the other hand, APD2 (http://aps.unmc.edu/AP/prediction/prediction_main.php) contains 2684 antimicrobial peptides from six kingdoms (266 from bacteria, 4 from archaea, 8 from protists, 13 from fungi, 329 from plants and 2018 from animals).

### Predicted peptides and mutant synthesis

The five predicted peptides (L1, L2, L8, L10 and L12) (Table 2) and three mutants (M1, M2 and M3) (Table 3) were synthesized by a commercial company (Scilight Biotechnology, Beijing, China) using the solid-phase procedure. Briefly, 9-fluorenylmethoxycarbonyl amino acid (Fmoc-amino acid) and 2,6-dichlorobenzenoylchloride (DCB) were added to the resin to attach the first amino acid. The deprotection was conducted by adding piperidine to remove the protection group, and then the activated amino acid was attached to the peptidyl resin with agitation to couple the next residue. The cycle of deprotection and coupling was repeated until the target peptide was completely synthesized. The peptide was then cleaved from the resin with TFA. The disulfide bridge in the peptide was formed by DMF, and C-terminal amidation was conducted by the amidating enzyme. The synthetic peptides were purified by HPLC with an Agela C18 column. The purity and molecular masses of the purified synthetic peptides were determined using liquid chromatography coupled to mass spectrometry (LC–MS/ESI).

### Minimal inhibitory concentration (MIC)

Seven bacteria including *Vibrio parahaemolyticus*, *Vibrio fluvialis*, *Vibrio alginolyticus*, *Esch*e*richia coli*, *Aeromonas hydrophila*, *Staphylococcus aureus* and *Streptococcus pyogenes* were grown in LB medium at 30 °C and diluted to 10^6^ CFU/mL in 0.01 M, pH 7.6 PBS. Peptides were diluted in PBS. The bacteria and diluted peptides were mixed in equal volumes, and then incubated at 30 °C for 2 h. At the end of the incubation, bacteria were plated on LB agar and incubated at 30 °C overnight for MIC determination. The lowest concentration of peptide that resulted in the highest level of inhibition was used to define the MIC. Experiments were carried out in triplicate and repeated at least three times. Data are presented as mean ± standard deviation. GraphPad prism 6 was used to analyze the data with the statistical significance determined by one-way ANOVA and Student’s *t* test, and all experiments were carried out in triplicate.

### Circular dichroism (CD) spectrophotometry

The peptide CD spectra were collected on a Jasco 810 spectropolarimeter (Jasco, Tokyo). The synthesized peptides were dissolved in 25 mM sodium dodecyl sulfate (SDS) to 0.05 mg/mL with a path length of 1-mm quartz cuvette and data were recorded from 190 to 250 nm. The CD spectra were measured at 250 nm, the scanning speed was 100 nm/min and data points were recorded at 1.0 nm intervals. The CD data were processed by the CDpro software.

### Prediction of 3D structure

The 3D structures of synthesized peptides were initially predicted with the online server SWISS-MODEL (http://swissmodel.expasy.org/) and edited on PyMol program.

### Nuclear magnetic resonance (NMR) analysis

Synthesized peptides (5 mg) were dissolved in dimethyl sulfoxide (DMSO) plus 150 mM deuterated sodium dodecyl sulfate (SDS) and 0.2 mM 4,4-dimethyl-4-silapentane-1-sulfonic acid (DSS) as an internal reference and adjusted to pH 5. Samples were loaded into 5 mm thin-walled glass NMR tubes. All experiments were conducted on a Bruker Avance III 600 MHz NMR spectrometer equipped with a TXI probe and analyzed with MestReNova program. The stability of the alpha helix structure mainly depends on intra-chain hydrogen bonds. The N–H of each amino acid forms hydrogen bonds with the C=O of the fourth amino acids in the front. Analysis of the HMBC spectra was used to find the hydrogen bonding between N–H and C=O.

### Scanning electron microscopy (SEM) analysis

Two bacteria suspensions (*V. parahaemolyticus* and *S. aureus*) at 10^6^ CFU/mL were treated with an equal volume of synthesized peptide at lethal doses of 500 μg/mL or 0.01 M pH 7.4 PBS for 2 h. Five replicates were pooled together, centrifuged at 2700*g* for 10 min and washed twice with 0.01 M pH 7.4 PBS. The samples were then fixed with 4% formaldehyde for 30 min before rinsing with deionized water. A series of ethanol solutions (35, 50, 75, 90, 95 and 100%) was used to perform sample dehydration, after which they were mounted on copper tapes and allowed to air dry for 2 days. The samples were finally sputter coated with platinum before imaging under a field emission scanning electron microscope (JEOL JSM-7400F, Japan) (Khara et al. 2015).

## Results

### Bioinformatics prediction of AMPs from shrimp hemocyanin

Hemocyanins are large, copper-containing, multi-subunit oxygen carrier proteins found in the hemolymph of both mollusks and arthropods. The large subunit of *L. vannamei* hemocyanin consists of 671 amino acids and is divided into three domains, including alpha helical region, copper-binding region and Ig-like region (Zhang et al. 2006).

**Fig. 1:**
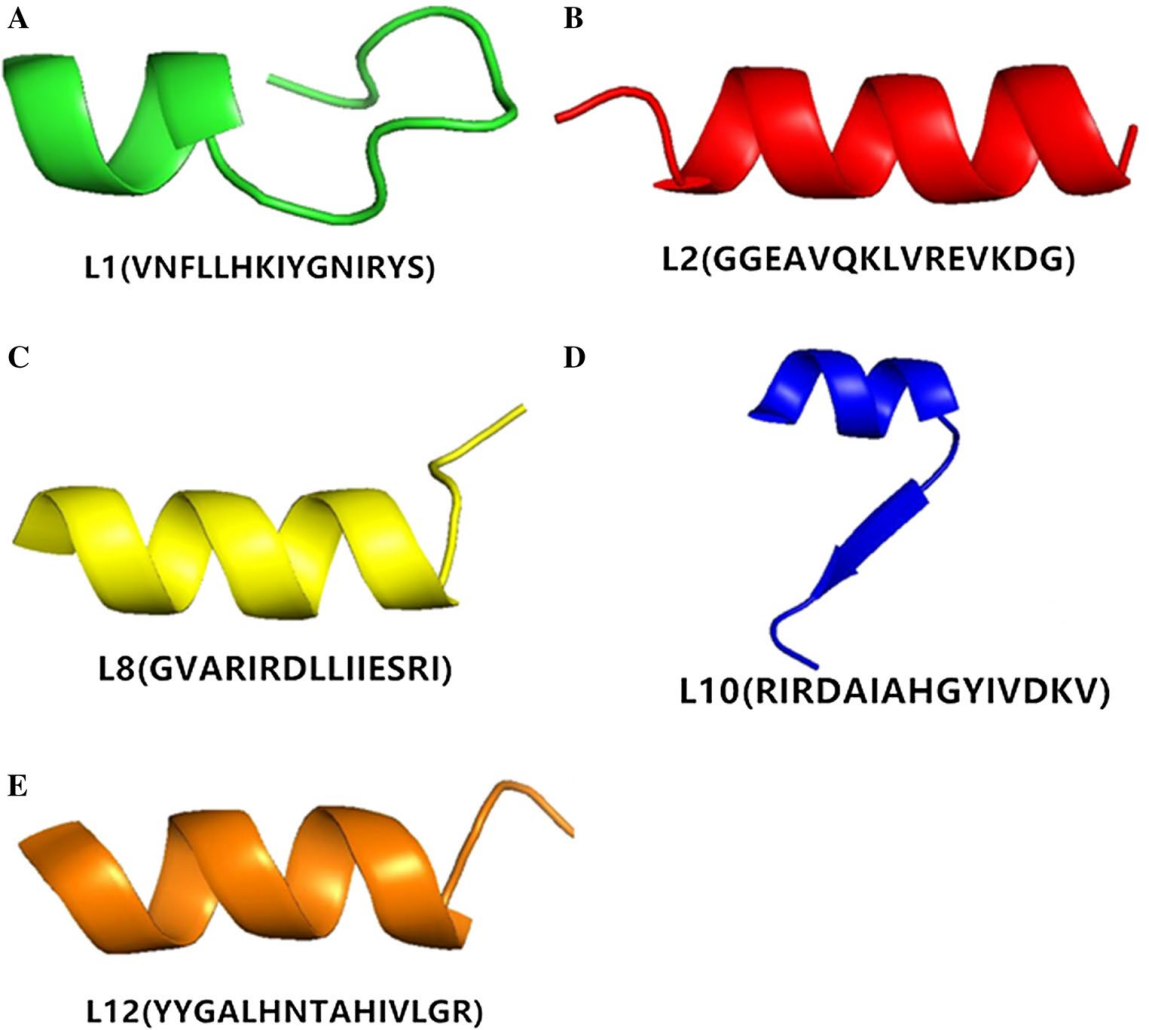
Five potential antimicrobial peptides of large subunit *L. vannamei* hemocyanin L1 (**a**), L2 (**b**), L8 (**c**), L10 (**d**) and L12 (**e**) predicted by APD2 software (http://aps.unmc.edu/AP/prediction/prediction_main.php)

First, two free online software packages (Soft AntiBP Server and CAMP) were used for the antimicrobial peptides prediction. Soft AntiBP Server was used to scan for active polypeptide fragments on the large subunit sequence of *L. vannamei* hemocyanin, while soft CAMP was applied to evaluate the credibility of the predicted active polypeptide fragments. As shown in Table 1, 20 predicted potential AMPs ranging from 1.5 to 1.9 kDa were predicted, among which 2, 10 and 8 came from domain I, domain II and domain III, respectively. Considering that a large number of known AMPs have secondary conformation structures such as α-helical (Zeth and Sancho-Vaello 2017; Takahashi et al. 2010) or β-strand (Yang et al. 2018; Wang et al. 2018), five of the predicted potential AMPs (L1, L2, L8, L10 and L12) had α-helical structures predicted by APD2 software and were selected for antibacterial activity testing (Fig. 1).

**Table 1:** Prediction of potential antibacterial peptides from large subunit *Litopenaeus vannamei* hemocyanin

No.	Peptide sequences	MW (Da)	Start position	Score	Antibacterial peptide probability	Domain	Alpha helical structure
L1	**VNFLLHKIYGNIRYS**	1837	32	0.068	0.741	I	Yes
L2	**GGEAVQKLVREVKDG**	1585	67	0.578	0.589	I	Yes
L3	KPGKFESSFTGTKKN	1649	180	0.368	0.767	II	No
L4	DDKYSHHLDRKGGNF	1788	224	0.661	0.678	II	No
L5	YSHHLDRKGGNFFWV	1863	227	0.216	0.641	II	No
L6	HHLDRKGGNFFWVHH	1887	229	0.234	0.826	II	No
L7	HLDRKGGNFFWVHHQ	1878	230	1.436	0.589	II	No
L8	**GVARIRDLLIIESRI**	1724	300	0.130	0.560	II	Yes
L9	RIRDLLIIESRIRDA	1839	303	0.596	0.771	II	No
L10	**RIRDAIAHGYIVDKV**	1726	313	1.147	0.677	II	Yes
L11	DAIAHGYIVDKVGNH	1608	316	0.002	0.517	II	No
L12	**YYGALHNTAHIVLGR**	1684	357	0.013	0.627	II	Yes
L13	GRWNAIELDKFWVKL	1875	515	0.398	0.511	III	No
L14	FWVKLPGGTHHIERK	1799	525	0.802	0.608	III	No
L15	WVKLPGGTHHIERKC	1755	526	0.641	0.565	III	No
L16	NHYGSHGVYPDKRPH	1751	621	0.016	0.759	III	No
L17	GSHGVYPDKRPHGYP	1648	624	0.648	0.624	III	No
L18	FGHIHLKVFNHGEHI	1785	655	0.698	0.699	III	No
L19	GHIHLKVFNHGEHIH	1775	656	0.064	0.746	III	No
L20	HIHLKVFNHGEHIHH	1855	657	0.353	0.637	III	No

Bold type signifies ‘Alpha helical structure’

*I* alpha helical domain, *II* copper-binding domain, *III* Ig-like domain

### Antibacterial analysis of five predicted potential AMPs

**Fig. 2:**
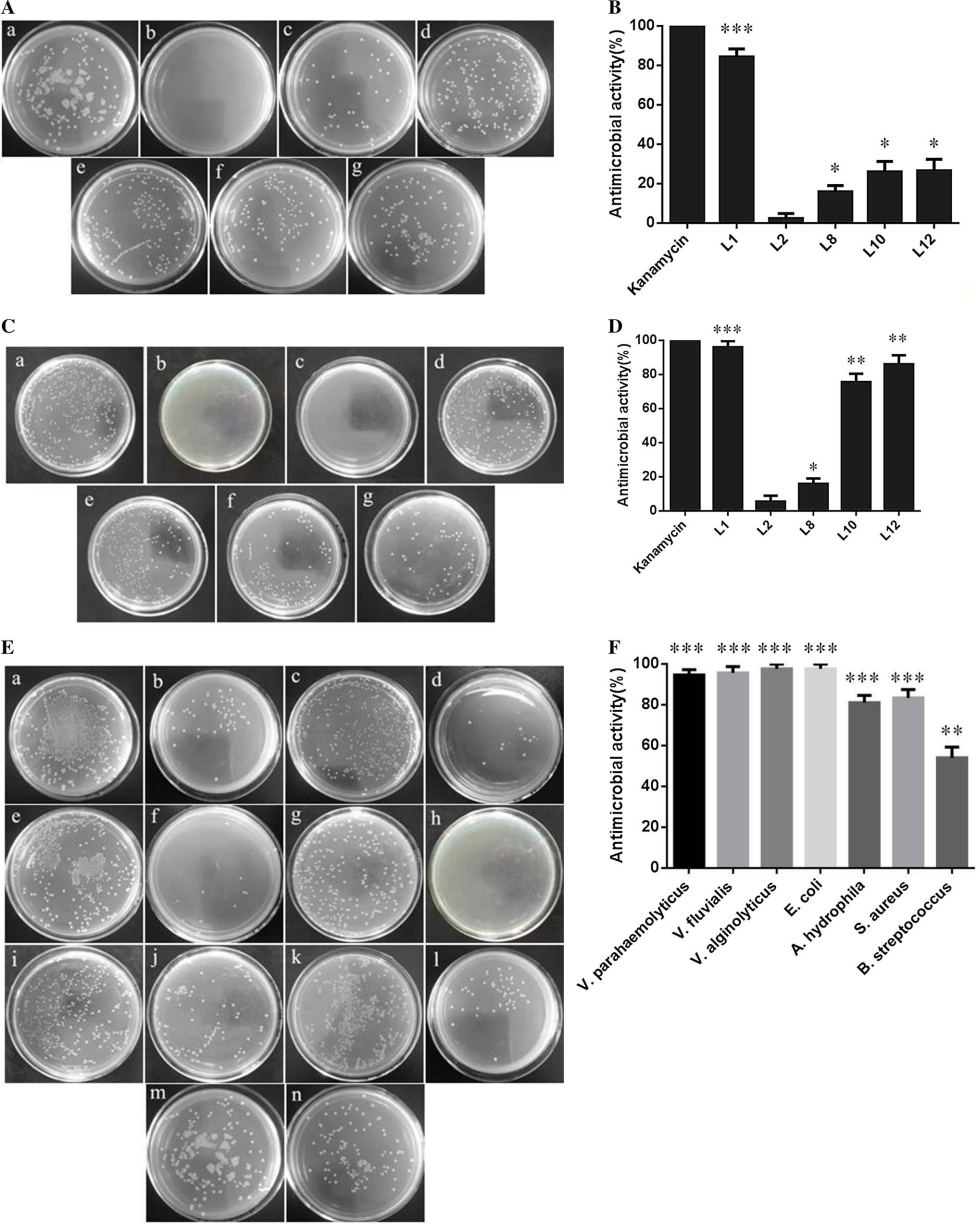
The antibacterial activity of five predicted potential AMPs. **a**, **c** Bacterial colonies in a Petri dish of 0.01 M pH7.4 PBS (a, negative control), kanamycin (b, positive control), peptide L1 (c), peptide L2 (d), peptide L8 (e), peptide L10 (f) and peptide L12 (g) with *V. alginolyticus* and *S. pyogenes*, respectively. **b**, **d** Antimicrobial activity of samples in **a** and **c**, respectively. **e** Bacterial colonies in a Petri dish of 0.01 M pH 7.4 PBS (a, c, e, g, i, k and m) or peptide L1 (b, d, f, h, j, l and n) with *V. parahaemolyticus* (a, b), *V. fluvialis* (c, d), *V. alginolyticus* (e, f), *E. coli* (g, h), *A. hydrophila* (i, j), *S. aureus* (k, l) and *S. pyogenes* (m, n), respectively. **f** Antimicrobial activity of samples in **e**. The concentration of the five peptides and kanamycin is 0.5 mg/ml. The experiments were carried out in triplicate, repeated at least three times and the significant difference was determined by one-way ANOVA relative to control and indicated by asterisks (*p < 0.05, **p < 0.01 and ***p < 0.001). Data are presented as mean ± SD. Error bars represent standard deviation (SD) for the three independent experiments

To validate whether the five predicted potential AMPs (L1, L2, L8, L10 and L12) had antibacterial activity, they were synthesized (by a commercial company) and their MICs measured with *V. parahaemolyticus*, *V. fluvialis*, *V. alginolyticus*, *E. coli*, *A. hydrophila*, *S. aureus* and *S. pyogenes*. The results indicated that the five predicted potential AMPs had various antibacterial activities against these different bacteria. Notably, L1 had the strongest antibacterial activity against both Gram-negative and Gram-positive bacteria, while L2 had the weakest antimicrobial activity against all seven bacteria. The average MIC of L2 was tenfold that of the average MIC of L1. The AMPs L8, L10 and L12 had some selective antimicrobial activities against these bacteria. For example, L8 and L10 showed moderate antimicrobial activity against *A. hydrophila*, while L10 and L12 showed moderate antimicrobial activity against *V. alginolyticus* (Table 2 and Fig. 2).

**Table 2:** Antimicrobial activities of the predicted potential AMPs

Peptides	MIC^a^ (μgmL^−1^)							GB^b^ (μgmL^−1^)
	Gram negative					Gram positive		
	*V. parahaemolyticus*	*V. fluvialis*	*V. alginolyticus*	*E. coli*	*A. hydrophila*	*S. aureus*	*S. pyogenes*	
L1	31.2	62.5	3.9	3.9	3.9	62.5	62.5	14.2
L2	250	125	125	> 250	250	> 250	250	205.1
L8	250	250	250	> 250	31.3	250	250	185.8
L10	250	125	31.3	> 250	31.3	> 250	125	113.3
L12	250	250	15.6	125	125	> 250	125	125.0
Kanamycin	1	1	0.5	2	1	2	1	1.1

### Structural analysis of L1

**Fig. 3:**
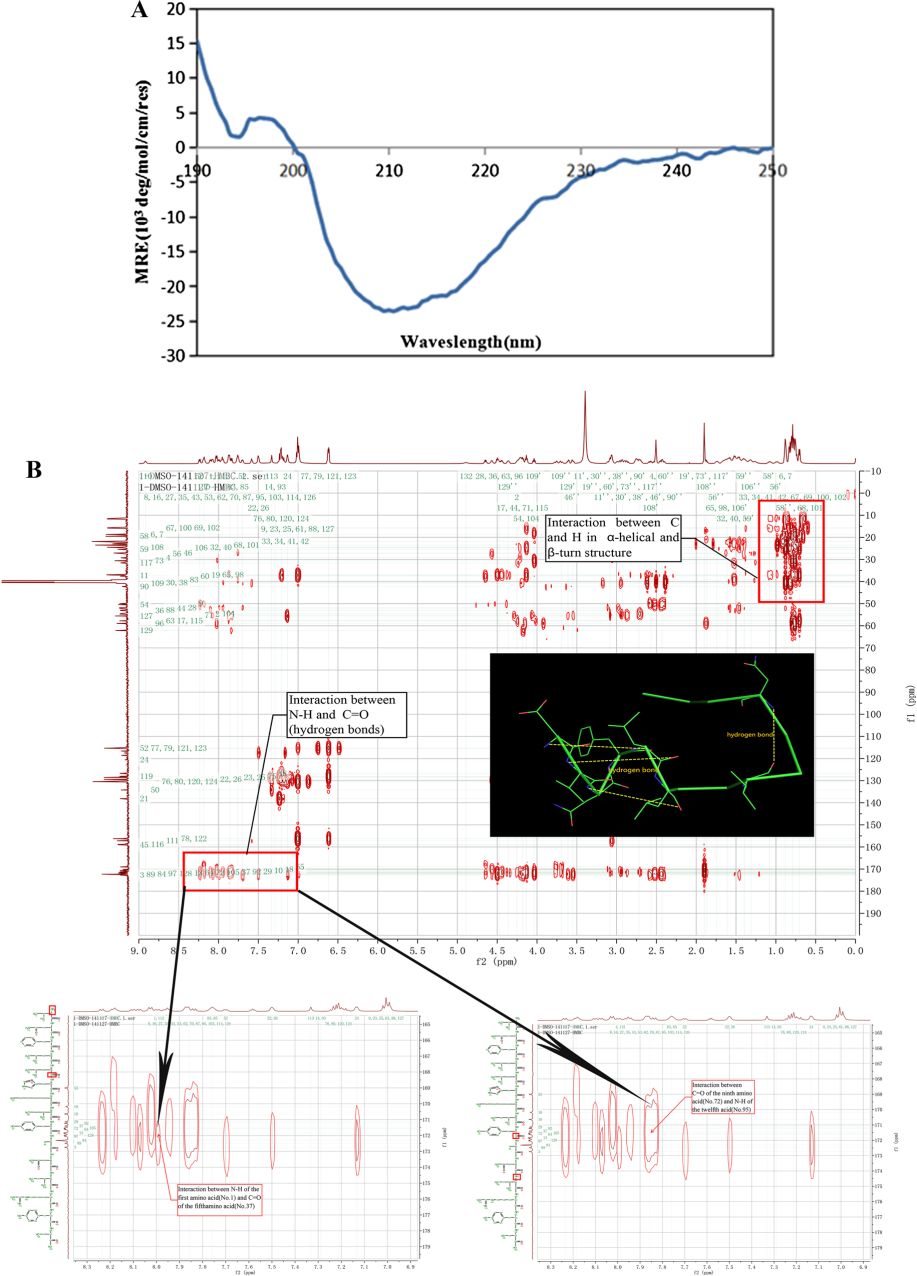

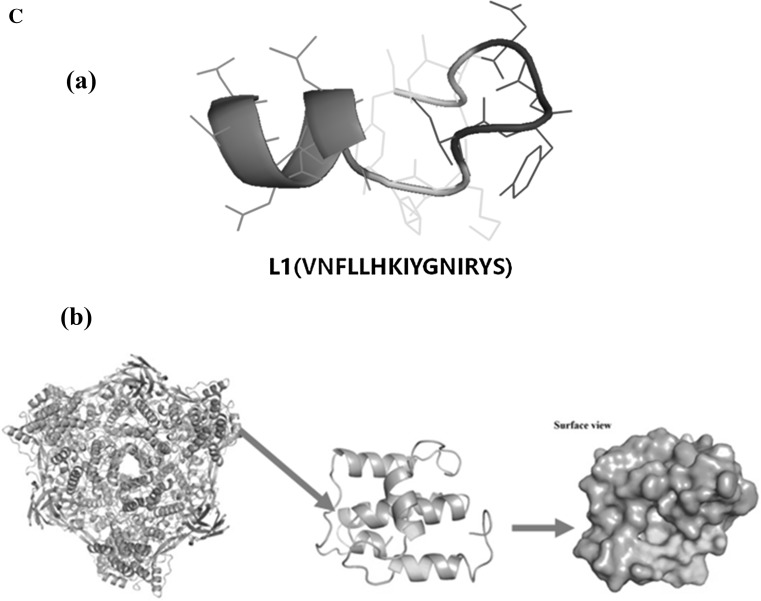
Structural analysis of peptide L1. **a** CD spectra of peptide L1 at 25 °C. **b** HMBC spectrum of peptide L1 in DMSO. **c** *In silico* model of peptide L1. (a) Structural views of large subunit *L. vannamei* hemocyanin hexamer and modeled peptide L1; green part represents L1 in the picture (b) (color figure online)

To elucidate the solution conformation of L1, circular dichroism (CD) experiments were performed. The results indicated that L1 had 34.6% α-helical, 11.2% β-strand, 24% β-turns and 33.4% random coil (Fig. 3a and Data Reports S1). Next, the secondary structure of L1 was determined using NMR. HMBC (heteronuclear multiple bond correlation) spectra were used for detailed structural analysis. As shown in Fig. 3b and Figure S1, the α-helical structure of L1 starts from amino acids 1–5, and N–H of each peptide bond could bind to C=O of the fourth peptide bond to form hydrogen bonds, with each peptide bond participating in hydrogen bond formation in the peptide chain. The β-turn structure starts from amino acids 8–11, and C=O of the first amino acid residue could bind to N–H of the fourth amino acid residue to form hydrogen bonds. The secondary structure of L1 is shown in Fig. 3c. Since our NMR data (analyzed with MestReNova software) was consistent with the CD data (processed by the CDpro software), it strongly suggests that the secondary structure composition of L1 peptide is correct.

### Analysis of the antibacterial mechanism of AMP L1

**Fig. 4:**
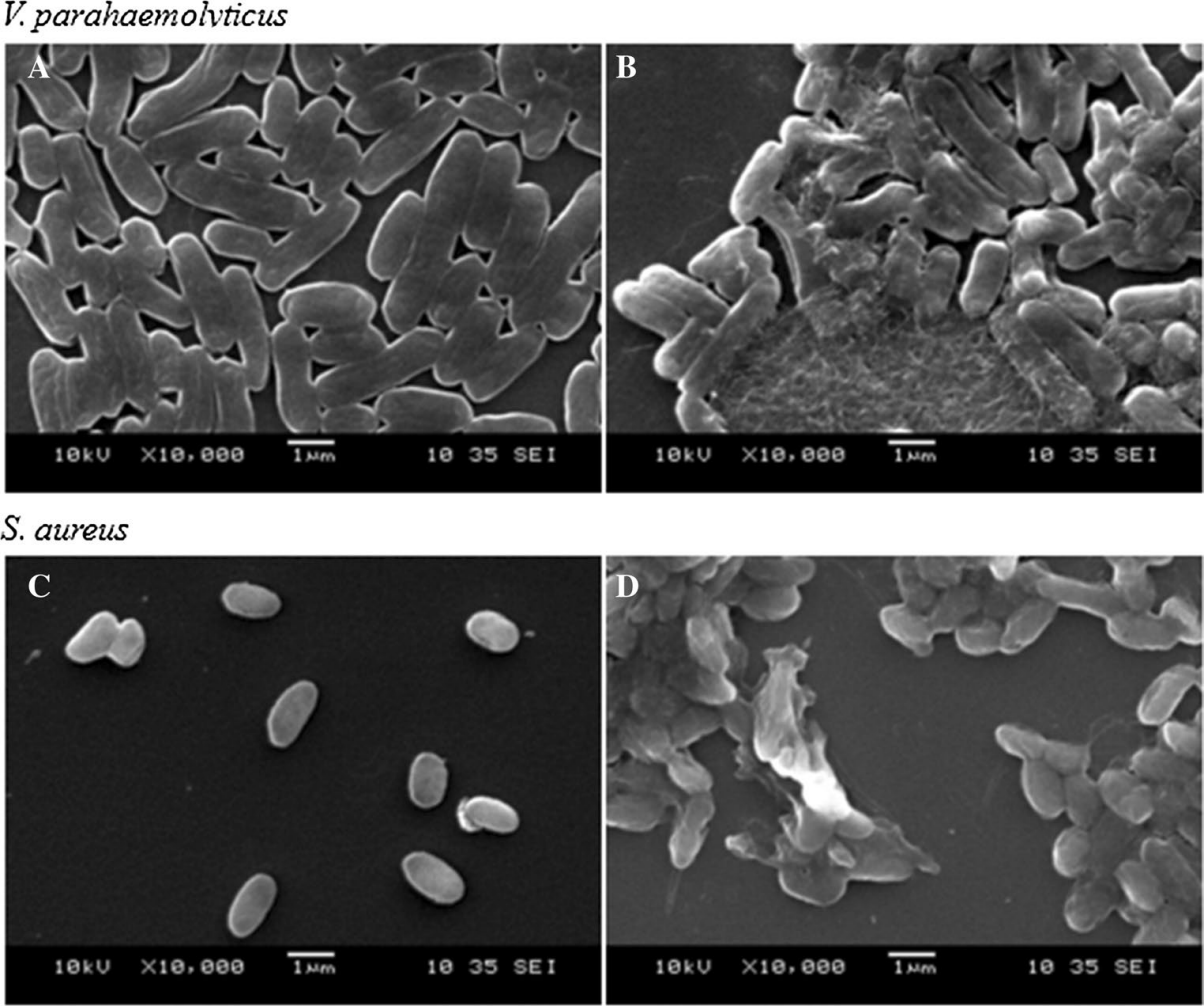
Scanning electron micrographs of *V. parahaemolyticus* (**a**, **b**) or *S. aureus* (**c**, **d**) with 0.01 M, pH7.4, PBS (**a**, **c**) and 500 μg/mL peptide L1 (**b**, **d**), respectively

Scanning electron microscopy (SEM) was applied to determine the antibacterial activity against two L1-treated bacteria, *V. parahaemolyticus* and *S. aureus*. It was observed that L1 treatment resulted in bacteria roughening and boundary fuzzing with some intracellular inclusions, which indicated that the bacterial cell walls were destroyed, with the degraded polysaccharides aggregating to form a agglomerate structure (Fig. 4).

To further examine the relationship between L1 secondary structure and its antimicrobial activity, different L1 mutants including M1 (Gly to Phe substitution to destroy the α-helical structure), M2 (Asp to Gly substitution to mutate the β-turn structure), and M3 (Asp to Try substitution as control) were designed and synthesized. The results showed that the antibacterial activities of M1 and M2 were decreased 2- to 64-fold (except for *S.pyogenes*), while no change was observed to that of M3, which indicated that α-helical and β-turn were essential conformations for L1’s antibacterial activity (Table 3).

**Table 3:** Antimicrobial activities of peptide L1 and L1 mutant peptides

Peptides	MIC^a^ (μgmL^−1^)							GB^b^ (μgmL^−1^)
	Gram negative				Gram positive			
	*V. parahaemolyticus*	*V. fluvialis*	*V. alginolyticus*	*E. coli*	*A. hydrophila*	*S. aureus*	*S. pyogenes*	
VNFLLHKIYGNIRYS (L1)	31.2	62.5	3.9	3.9	3.9	62.5	62.5	14.2
VN**G**LLHKIYGNIRYS (M1)	62.5	250	250	15.6	15.6	> 250	15.6	84.3
VNFLLHKIY**D**NIRYS (M2)	62.5	> 250	> 250	7.8	15.6	> 250	62.5	69.0
VNFLLHKIYGNIR**D**S (M3)	31.2	62.5	3.9	3.9	3.9	62.5	62.5	14.2

Bold type signifies mutation site

## Discussion

Previous studies have shown that hemocyanin exerted its antimicrobial function based on different mechanisms including glycosylation modification (Zhang et al. 2017) and molecular polymorphism (Zhao et al. 2016; Lu et al. 2015; Guo et al. 2013; Zhao et al. 2012, 2013). Here, we predicted and characterized a novel hemocyanin-derived antimicrobial peptide L1 using bioinformatics, followed by experimental validation. The peptide L1 had broad-spectrum activity against both Gram-positive and Gram-negative bacteria. SEM analysis showed that L1 could destroy these bacteria, as their cell walls were seriously damaged with the efflux of intracellular content (Fig. 4), indicating that L1 could kill the bacteria. Peptide L1 is a cationic peptide which has two positively charged residues (Lys7 and Arg13). Although we did not perform any mutation on Lys7 and Arg13, some studies have reported that these positively charged amino acid residues (Lys and Arg) play an important role in the sequence of antimicrobial peptides (Ahn et al. 2006). In addition, the CD and NMR data showed that peptide L1 had a typical α-helix, which was similar to melittin, an antimicrobial peptide consisting of 26 amino acids (Renzo et al. 1988). Thus, the antimicrobial mechanism of L1 may involve electrostatic adsorption onto the cell membrane and form helical hydrophobic environment on the cell membrane. However, a clear-cut mechanism on this needs to be explored in further studies.

Currently, AMPs can be divided into three subgroups based on their secondary structure (Zasloff 2002; Epand and Vogel 1999): (1) α-helical antimicrobial peptides (Durr et al. 2006; Pandey et al. 2010), including α-defensins, cecropins, magainins, melittin and piscidins; (2) β-sheets antibacterial peptides, including cyclic β-sheets, two or more disulfide bonds consisting of β-sheets (Salgado et al. 2001; Brogden 2005), such as gramicidins, β-defensins, tachyplesins, protegrins, thionin and polymyxin; (3) random coil antibacterial peptides, such as tritrpticin in the family of cathelicidin (Andrushchenko et al. 2007). Given that replacement of Phe with Gly in L1 could effectively destroy its α-helical structure as well as its antibacterial activity (Table 3), it thus implied the α-helical structure was the functional domain of peptide L1, which is synonymous with previous studies (Deslouches et al. 2005). Similarly, when the Gly10 was replaced with Asp, the β-turn structure of peptide L1 was destroyed, which also caused a sharp decrease in the antimicrobial activity of L1 (Table 3), therefore suggesting that the β-turn of L1 could also be the functional domain of this antibacterial peptide. A similar observation was also previously reported by Park et al. (2011). Thus, based on the mutational analysis of L1, it could be classified as an α-helical β-turn antimicrobial peptide (Yang et al. 2002).

While the peptide L1 had the strongest broad-spectrum antimicrobial activity, a few of the other peptides identified in the screen also had antibacterial activities against some of the tested bacteria. For instance, among the five peptides, which had α-helical structures (Fig. 1), L8 had antibacterial activity on *A. hydrophila*, L10 on *V. alginolyticus* and *A. hydrophila*, while L12 had antimicrobial activity on *V. alginolyticus*. One other noteworthy finding in this study was that there was some level of correlation between the predicted antimicrobial activity and the experimentally measured minimal inhibitory concentration (MIC) of the peptides. Peptide L1 had the lowest MIC and the highest antimicrobial peptide probability (0.741), while L12 and L10 had medium MIC and antimicrobial peptide probability of 0.627 and 0.677, respectively. On the other hand, L8 and L2 had a high MIC and the lowest antimicrobial peptide probability of 0.560 and 0.589, respectively. It is therefore plausible to infer that the capability of bioinformatic tools to provide semi-quantitative prediction on MIC is high, and therefore could shorten or speed up the discovery of AMPs or small molecules. Moreover, this approach in rational design of antimicrobial peptides is much more efficient than the traditional approach, and hence can be optimized for large-scale use. For the peptides identified in this study, further experiments would have to be carried out so as to give a better insight into their mode of action. Until now, most of the hemocyanin-derived peptides have been derived from other species. The AMPs from these other species are generated from hemocyanin at sites distant from the copper-binding domain, because their metal binding sites are mostly hydrophilic. Apart from this, most of the known AMPs are usually found within the C-terminal domain (III) of hemocyanin subunits (Lee et al. 2003; Destoumieux-Garzon et al. 2001; Qiu et al. 2014; Coates and Nairn 2014), because there is an Ig-like conserved domain of 252 amino acid residues in the C-terminus of arthropods hemocyanins, which is structurally homologous with human Ig (Zhang et al. 2006, 2017). In the present study, peptide L1 was found to be located in the N-terminal domain (I) (Fig. 3c), which means that the N-terminal domain (I) of large subunit *L. vannamei* hemocyanin could be a new immune region.

## Conclusion

In conclusion, five potential hemocyanin-derived AMPs were predicted using bioinformatics, followed by antibacterial analysis. Of these, peptide L1 had the strongest antibacterial activity and could be classified as α-helical β-turn antimicrobial peptide, which is capable of destroying the bacterial membrane by forming an agglomerate structure. This work could provide a more efficient approach to explore some novel AMPs and useful information for the investigation of the shrimp immune defense mechanisms.

## Electronic supplementary material

Below is the link to the electronic supplementary material.

Supplementary material 1 (DOC 71 kb)

Supplementary material 2 (DOC 1009 kb)
