# Supplementary material for: Prediction and characterization of a novel hemocyanin-derived antimicrobial peptide from shrimp Litopenaeus vannamei
Source: Amino Acids. 2018 May 4;50(8):995–1005. doi: 10.1007/s00726-018-2575-x (PMC6060862; doi:10.1007/s00726-018-2575-x)
Supplement: Supplementary file 1 — Supplementary material 1 (DOC 71 kb) [file 726_2018_2575_MOESM1_ESM.doc]

**Data Reports S1.** The CD Data report of AMP L1

THE FRACTIONS OF SECONDARY STRUCTURES FROM THE SELF-CONSISTENT METHOD

Sreerama and Woody, Anal. Biochem. (1993), 209, 32

Sreerama and Woody, Biochemistry, 33, 10022-25, (1994),

Sreerama et al. Protein Science, 8, 370-380, (1999),

Johnson W.C.Jr.,Proteins:Str.Func.Genet.35,307-312(1999)

BASIS SET OPTIONS AVAILABLE

| **IBasis** | **Ref.Set** | **Proteins** | **Secondary Structre** | **WaveLen** |
| --- | --- | --- | --- | --- |
| 1 | SP29 | 29-SOLUBLE | H1,H2,S1,S2,T,U | 178-260 |
| 2 | SP22X | 22-SOLUBLE | H,3-10,S,T,P2,U | 178-260 |
| 3 | SP37 | 37-SOLUBLE | H1,H2,S1,S2,T,U | 185-240 |
| 4 | SP43 | 43-SOLUBLE | H1,H2,S1,S2,T,U | 190-240 |
| 5 | SP37A | 37-SOLUBLE | H,S,P2,T,U | 185-240 |
| 6 | SDP42 | SP37,5 Denatrd | H1,H2,S1,S2,T,U | 185-240 |
| 7 | SDP48 | SP43,5 Denatrd | H1,H2,S1,S2,T,U | 190-240 |
| 8 | CLSTR | Soluble/Denatr. | H1,H2,S1,S2,T,U | 190-240 |
| 9 | SMP50 | SP37,13 Membran | H1,H2,S1,S2,T,U | 185-240 |
| 10 | SMP56 | SP43,13 Membran | H1,H2,S1,S2,T,U | 190-240 |

Reference PROTEIN Set Selected: SMP56

56 Proteins- 43 Soluble and 13 Membrane

Structures: Helix1, Helix2, Strand1, Strand2, Turns and Unordered

Total Helix content = Helix1 + Helix2

Total Sheet content = Strand1 + Strand2

NUMBER of Segments Calculated

SUMMARY in ProtSS.out

Contains Sec. Str. Fractions, RMSD and NRMSD

RMSD and Normalized-RMSD between Exp and Calc CD spectra

NRMSD from the three programs generally vary in the order:

CONTINLL < CDSSTR < SELCON3

Use CAUTION in drawing CONCLUSIONS from RMSD/NRMSD

L1

The wavelength range: 250.0 190.0, is different from that of

the Database proteins: 240.0 190.0

The No. of CD points = 61

The wavelengths are modified to be within the valid range

Beginning wavelength: 240.00; Ending wavelength: 190.00

Total No of CD points 51

Beginning wavelength: 240.00; Ending wavelength: 190.00

Total No of CD points 51

Secondary structural elements= 6

Multipilcation FACTOR for CD spectrum= 1.000

**SAMPLE CD: Protein - L1**

-1.56 -2.34 -2.96 -3.40 -4.16 -4.41 -4.54 -5.71 -6.19 -6.97 -7.62 -8.24 -8.89 -8.99 -8.97 -9.29 -9.82 -9.94 -10.18 -10.84 -11.12 -11.06 -11.71 -11.63 -11.32 -10.93 -10.28 -9.77 -8.73 -7.84 -6.19 -5.72 -5.21 -3.81 -3.09 -2.20 -2.95 -6.09 -7.96 -9.33 -11.38 -12.93 -11.34 -8.98 -5.70 -3.47 1.73 5.50 8.53 12.87 15.94

Number of Proteins in the database= 57

The SAMPLE data for: L1

**Ordered DELTA(CD) values :**

0.0000 5.9951 6.1985 6.2396 6.4615 6.5298 6.5776 6.5908

6.7497 6.8051 6.8909 6.9158 6.9733 7.0004 7.0188 7.0283

7.0376 7.0681 7.0832 7.1888 7.2019 7.2075 7.2438 7.2929

7.3268 7.3375 7.3669 7.4940 7.5163 7.5535 7.5921 7.6012

7.6327 7.6429 7.7685 7.7717 7.7881 7.8075 7.9485 7.9676

8.0231 8.0234 8.0275 8.0578 8.1010 8.1771 8.2232 8.2425

8.3056 8.5736 8.6300 8.6360 8.7057 8.8974 9.0347 9.5518

9.7864

**Ordered List of PROTEINS** :

L1-0 PGK BPTI PAPN LYSM NUCL ECOR GRS

GPD CGA SUBB RNAS CYTC RHD RC_V LDH

ADK CHYT PARV CANH BLAC TPI SUDS FLVD

PS_I ColA SUBN ELAS THML PPSN CA2+ ADH

AntC INSL CPAS T4LS IFBP PRAL RC_S CyCO

AZU BTOX P_Cp HMRT HBN TNF CONA MGLB

GFP PhoE BNJN UCCR Rhod GCR OMPF BRho

LamB

IGUESS = 0; The structure of the Protein with closest CD spectrum: PGK

Initial Guess: 0.210 0.135 0.043 0.067 0.231 0.313

Solution Corresponding to All proteins in the Basis and 5 SVD vectors

This corresponds to Hennessey and Johnson Method

H(r) H(d) S(r) S(d) Trn Unrd SUM

-0.022 0.148 1.038 0.552 0.880 1.347 3.942

Helix From H & J Method: 0.125

FIRST STAGE:

Solutions With FIRST TWO Selection Rules:

This is for the First 19 Iterations only

Constraints relaxed to get a MIN # of Solns

TOTAL LL SOLUTIONS: 401

ITER: 1; AVE OF 50 SOLN: 0.219 0.132 0.054 0.069 0.228 0.323

SUM of SECNDRY STRUCTURE: 1.026

RMSD with Previous Guess: 0.0074

ITER: 2; AVE OF 17 SOLN: 0.244 0.134 0.063 0.063 0.211 0.299

SUM of SECNDRY STRUCTURE: 1.013

RMSD with Previous Guess: 0.0164

ITER: 3; AVE OF 26 SOLN: 0.253 0.132 0.069 0.061 0.206 0.297

SUM of SECNDRY STRUCTURE: 1.018

RMSD with Previous Guess: 0.0051

ITER: 4; AVE OF 24 SOLN: 0.260 0.130 0.074 0.060 0.202 0.294

SUM of SECNDRY STRUCTURE: 1.020

RMSD with Previous Guess: 0.0040

SOLN. CONVERGED: 0 ITERATIONS

MinSol: 1 SUM < 0.050

Solution at the END of FIRST STAGE:

H(r) H(d) S(r) S(d) Trn Unrd SUM

0.260 0.130 0.074 0.060 0.202 0.294 1.020

First Part Completed. The results roughly correspond

to SELCON and SELCON1 with only 2 selection rules

SECOND STAGE:

SOLUTIONS THAT SATISLY THREE SELECTION RULES:

I NSol Bas NS H(r) H(d) S(r) S(d) Trn Unrd SUM RRcn RExp

1 12 8 5 0.253 0.131 0.070 0.062 0.206 0.297 1.018 0.091 1.933

2 17 9 5 0.256 0.133 0.075 0.064 0.209 0.307 1.044 0.091 2.391

3 18 9 6 0.256 0.132 0.075 0.064 0.209 0.307 1.043 0.072 2.389

4 23 10 5 0.257 0.134 0.075 0.064 0.209 0.308 1.047 0.091 2.389

5 24 10 6 0.255 0.131 0.075 0.064 0.207 0.305 1.038 0.072 2.377

6 25 10 7 0.256 0.132 0.075 0.064 0.209 0.305 1.041 0.069 2.391

7 354 52 1 0.313 0.170 0.083 0.054 0.169 0.253 1.041 7.118 0.113

8 362 53 1 0.310 0.165 0.078 0.052 0.164 0.242 1.010 7.118 0.027

9 370 54 1 0.309 0.165 0.079 0.052 0.164 0.243 1.012 7.118 0.022

10 378 55 1 0.305 0.163 0.084 0.053 0.164 0.241 1.010 7.118 0.015

11 386 56 1 0.302 0.155 0.079 0.049 0.155 0.224 0.965 7.118 0.150

12 394 57 1 0.295 0.152 0.084 0.050 0.153 0.222 0.956 7.118 0.215

TotSOL > 1

Limits: ABS(Sum-1.0) < 0.050; Each Fraction > -0.030

RmsCD(Exp,Cal) < 0.250

SOLUTION AT SECOND STAGE (SELCON2):

Average Solution From 12 Solutions:

H(r) H(d) S(r) S(d) Trn Unrd SUM RRcn

0.281 0.147 0.078 0 .058 0.185 0 .271 1.019 4.695

Second Part Completed. The results roughly correspond SELCON2 with three selection rules
