# Supplementary material for: Prediction and characterization of a novel hemocyanin-derived antimicrobial peptide from shrimp Litopenaeus vannamei
Source: Amino Acids. 2018 May 4;50(8):995–1005. doi: 10.1007/s00726-018-2575-x (PMC6060862; doi:10.1007/s00726-018-2575-x)

**Figure S1** NMR（A）and HMBC（B）spectrum of AMP L1 in DMSO

**A**

**H Spectrum**


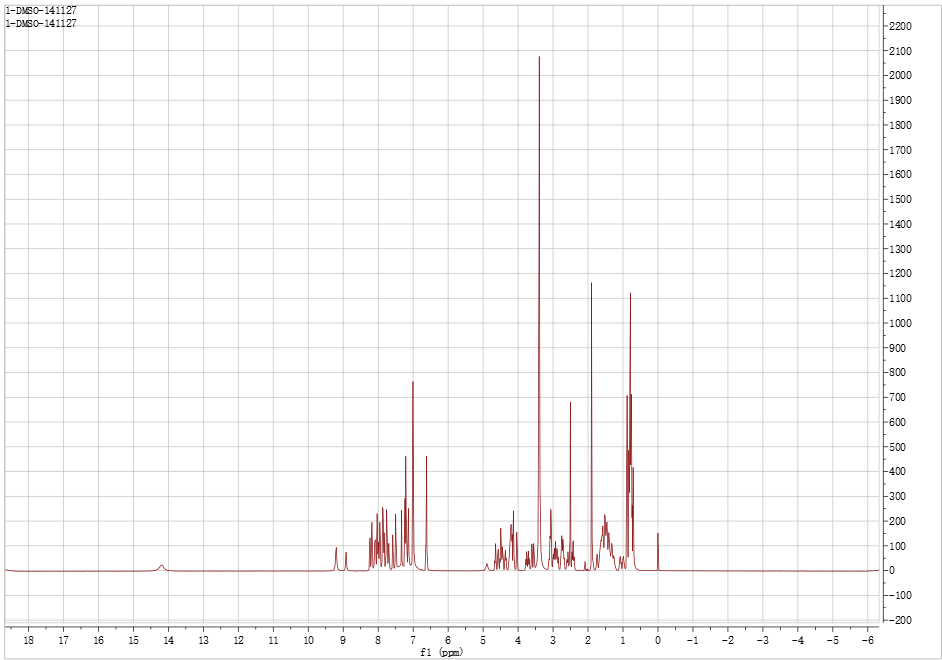


**C Spectrum**


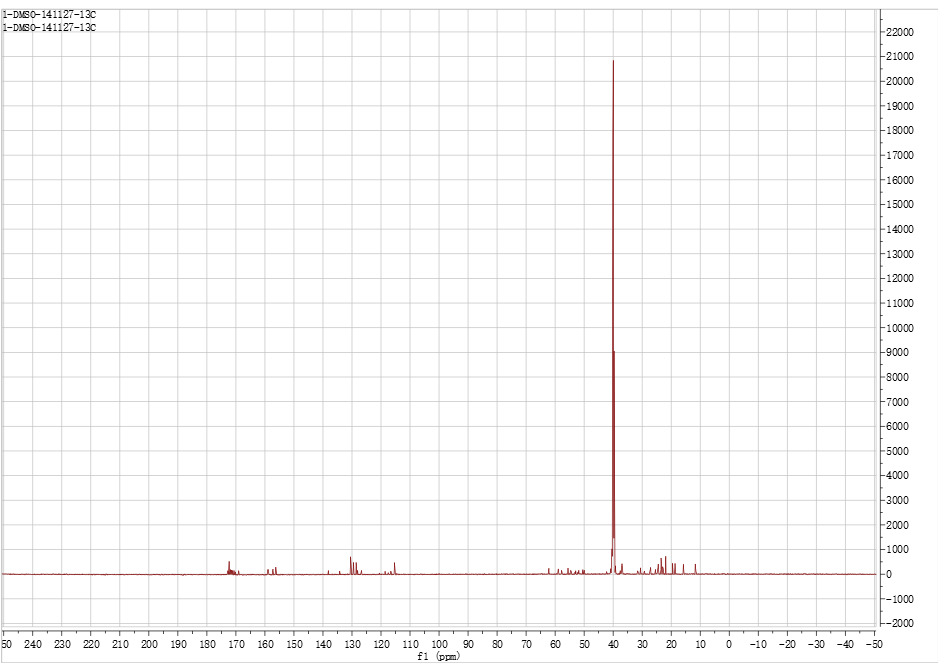


**COSY Spectrum**


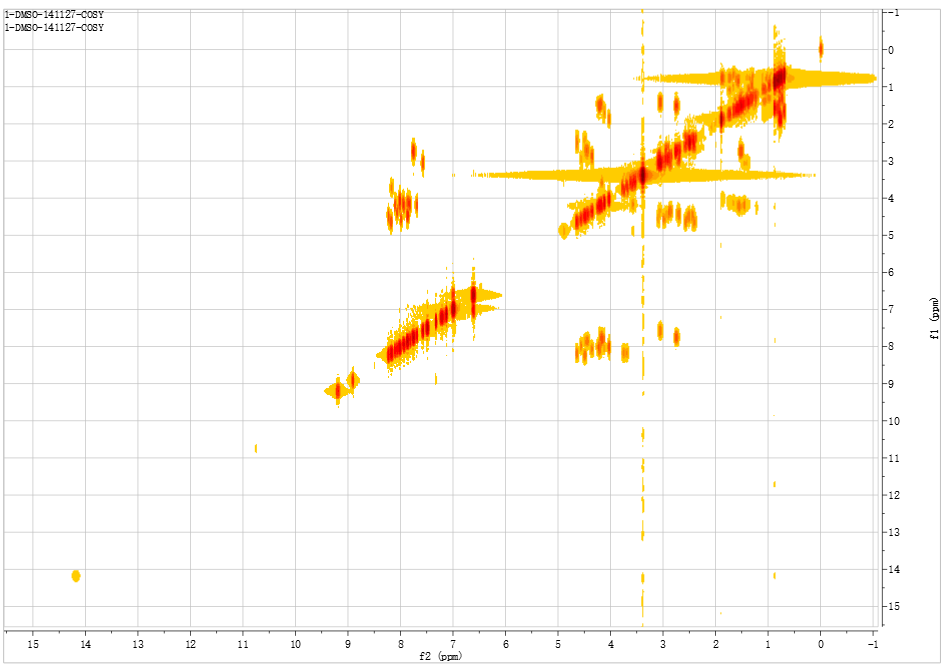


**B**

**HMBC**


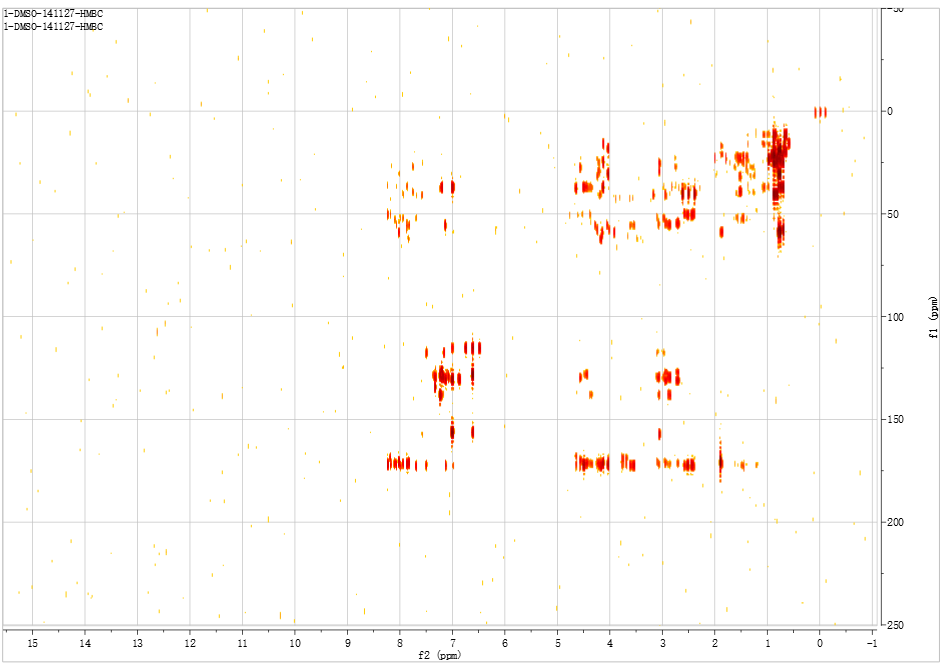


**HSQC Spectrum**


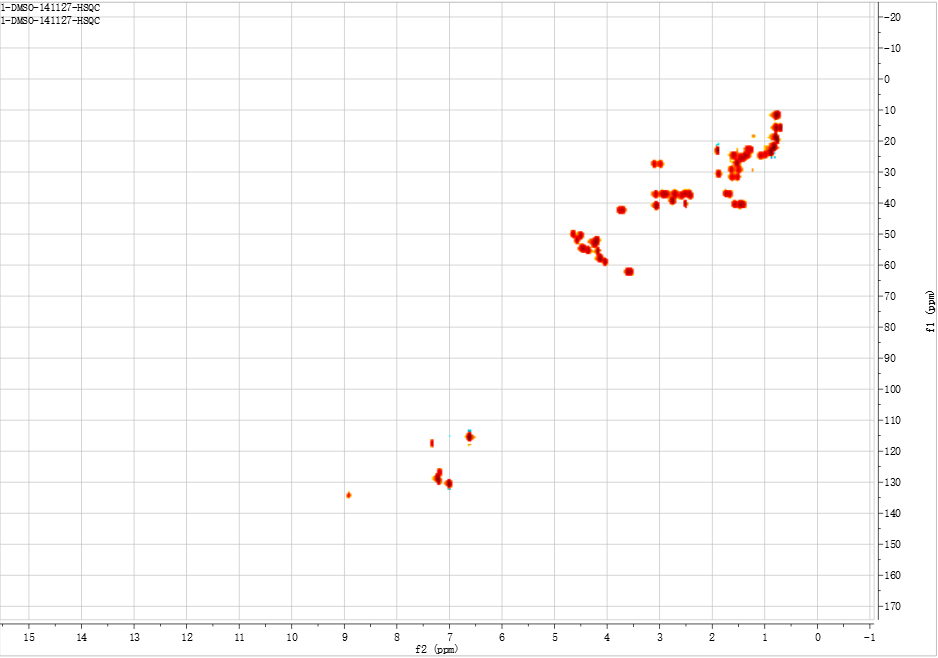


**NOESY Spectrum**


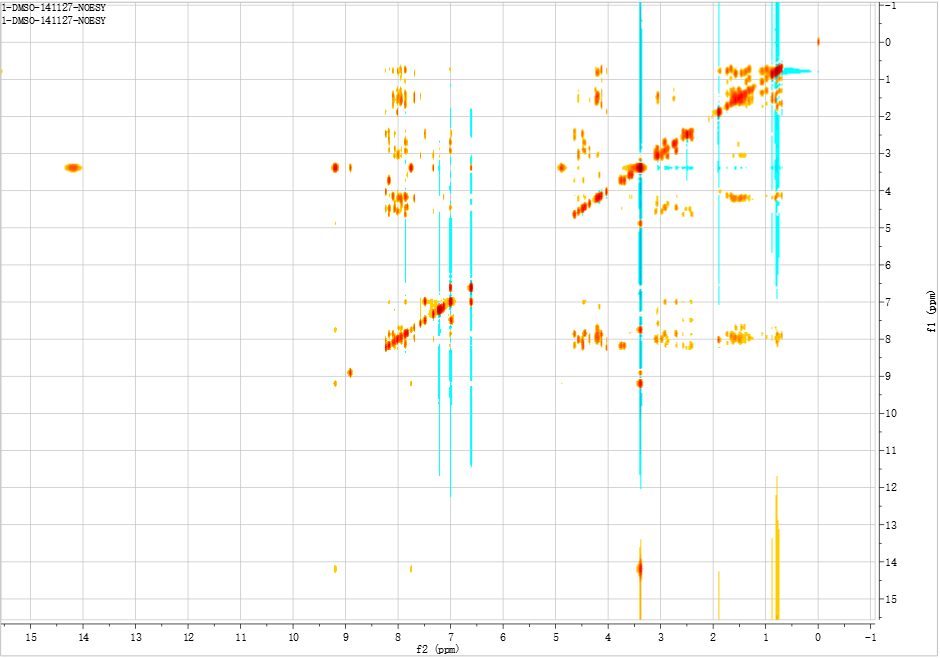


**TOCSY Spectrum**


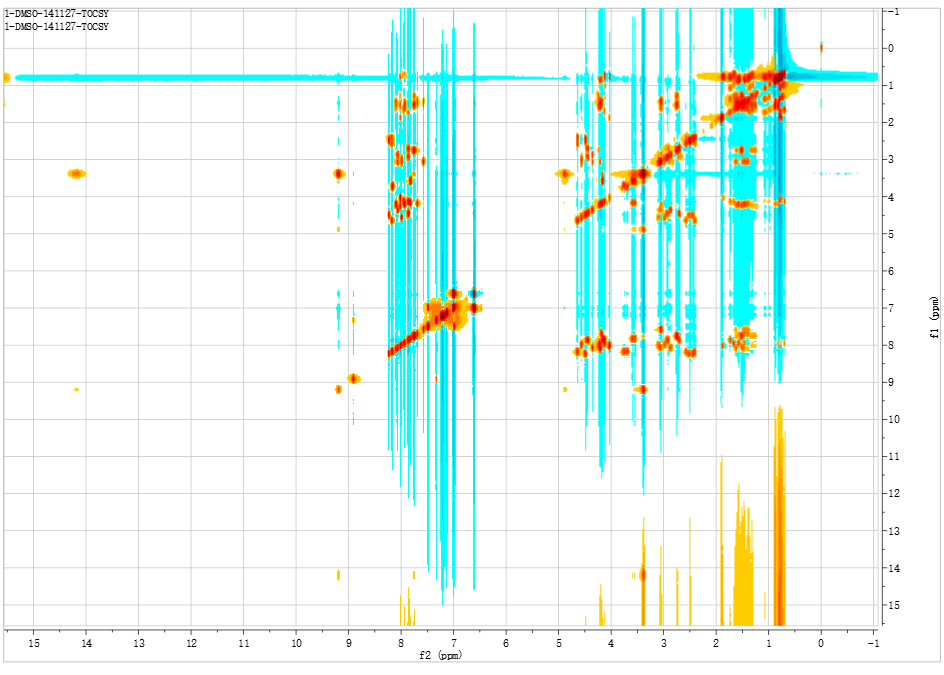


**Figure S2** **High Resolution Figures in Fig.3 B**


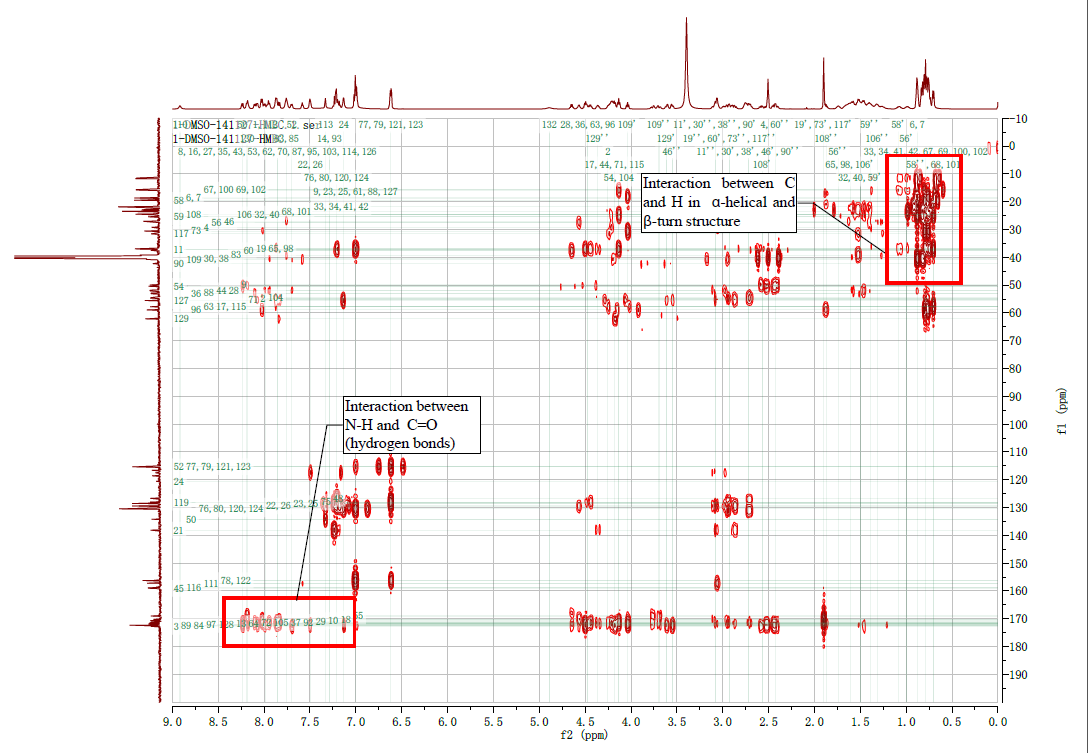


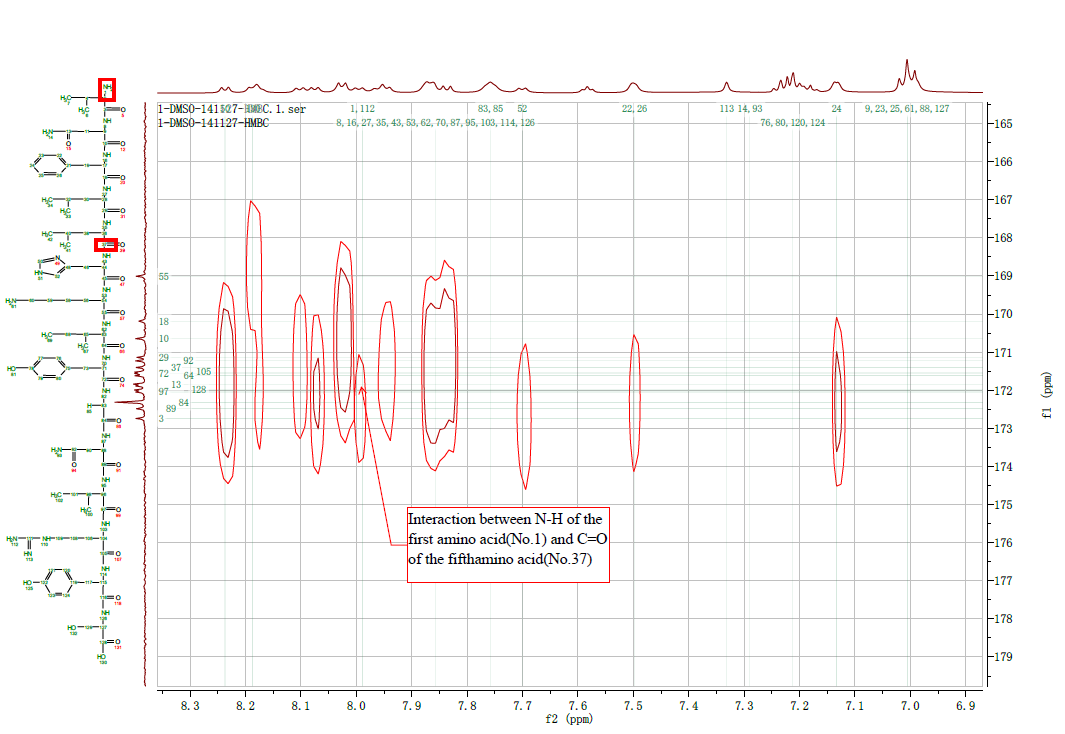


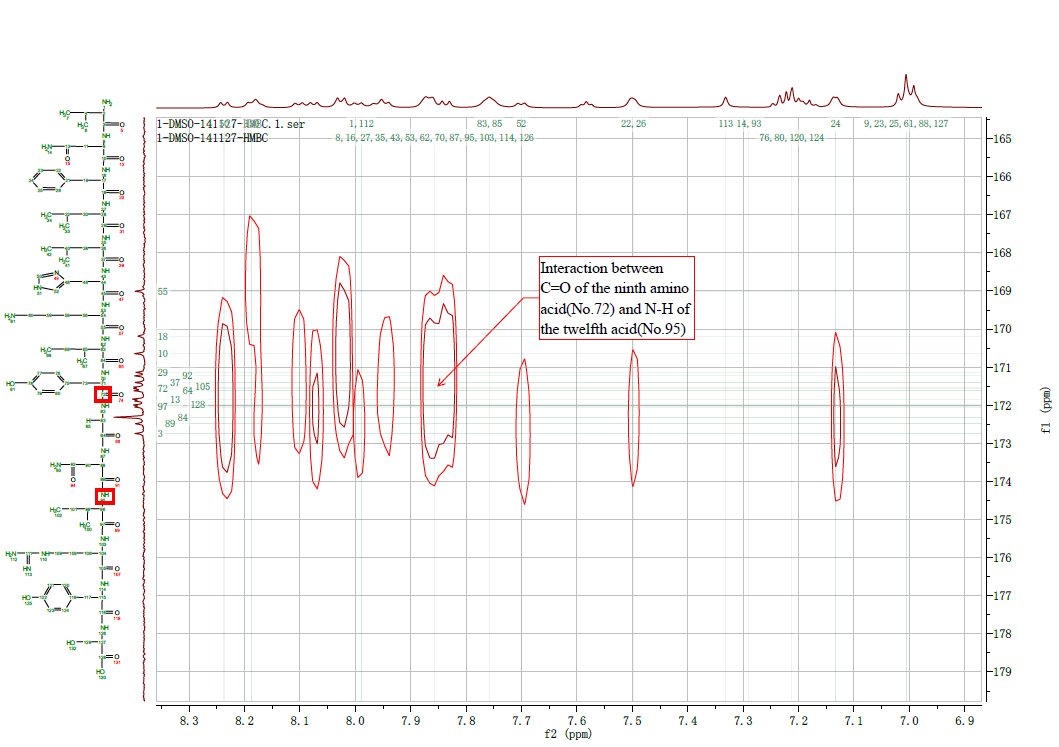

Supplement: Supplementary file 2 — Supplementary material 2 (DOC 1009 kb) [file 726_2018_2575_MOESM2_ESM.doc]
